# Potential of Z-100, extracted from *Mycobacterium tuberculosis* strain Aoyama B, as a hot tumor inducer

**DOI:** 10.1186/s12935-022-02821-6

**Published:** 2022-12-09

**Authors:** Takayuki Horii, Yuki Orikawa, Yuta Ohira, Runa Eta, Nobuyoshi Kobayashi, Takanori Sato, Takeshi Watanabe, Takao Tanaka

**Affiliations:** Central Research Laboratories, Zeria Pharmaceutical Co., Ltd., 2512-1, Numagami, Oshikiri, Kumagaya, Saitama 360-0111 Japan

**Keywords:** Antitumor effect, CD8^+^ T cell, Tumor-infiltrating lymphocytes, IL-12, Hot tumor

## Abstract

The tumor microenvironment is one of the most important factors determining the efficacy of cancer immunotherapy. In particular, variability in efficacy has been linked to whether tumors are hot or cold, with hot tumors exhibiting greater T cell infiltration and responding better to immunotherapy. Z-100 extracted from *Mycobacterium tuberculosis* Aoyama B strain has been reported to increase cytokine production from immune cells. In this study, we examined its effect on the tumor microenvironment and its potential as a hot tumor inducer. The antitumor effect of Z-100 was confirmed in a mouse oral squamous cell carcinoma (Sq-1979) tumor model by starting administration before tumor injection. Treated tumors were collected to identify infiltrating CD8^+^ T cells. The antitumor effects of Z-100 were additionally examined in mice treated with anti-CD8 antibody and in IL-12p40 knockout (KO) mice. We found that Z-100 had strong antitumor effects and increased the proportion of CD8^+^ T cells in tumors. Moreover, the CD8^+^ T cells infiltrating tumors were identified as effector memory CD8^+^ T cells. Furthermore, the antitumor effects of Z-100 were abolished in mice treated with an anti-CD8 antibody and in IL-12p40 KO mice. Thus, Z-100 induces its antitumor effects by increasing tumor-infiltrating CD8^+^ T cells, suggesting that Z-100 may be a useful cancer therapy by acting as a hot tumor inducer.

**Supplementary Information:**

The online version contains supplementary material available at 10.1186/s12935-022-02821-6.

## Introduction

The increasing clinical use of immunotherapy over recent years has made it clear that its efficacy varies among patients [1, 2]. One explanation for this could be related to the classification of tumors into hot or cold based on the tumor microenvironment, although this clinical subdivision of tumors into just two categories is somewhat controversial [3, 4]. Hot tumors reportedly have more T cell infiltration and respond better to immunotherapy [3, 4], while cold tumors have less T cell infiltration and respond worse to immunotherapy. Therefore, many oncologists are eager to find a way to turn tumors into hot tumors [5, 6].

Z-100 is a hot-water extract of human-type *Mycobacterium tuberculosis* strain Aoyama B that is clinically marketed for the treatment of leukopenia in Japan (Ancer^®^). Several reports have also described its antitumor effects [7–11]. According to a handful of studies on the mechanism of Z-100, it exerts its effects by inducing cytokines [10, 12, 13]. In particular, Z-100-mediated production of IL-12p40 from macrophages is thought to contribute to the drug’s anti-cancer effects [10, 12, 13]. A recent clinical study by Kobayashi et al. reported that combined use of Z-100 with an anti-PD-1 antibody was effective for treating lung cancer [14]. While the efficacy of Z-100 in combination with anti-PD-1 antibody suggests that Z-100 may have some effect on the tumor microenvironment, no reports have specifically examined this.

To study the effects of Z-100 on the tumor microenvironment, we first needed to identify the in vivo conditions under which Z-100 would have a marked effect on tumors. In previous reports, Z-100 was administered to tumor models immediately after tumor injection or a few days after tumor injection, but it has never been initiated prior to tumor injection [8–10]. However, the effect of clinical immunotherapy is known to be slow because it takes time for the immune environment to become established [15, 16]. Therefore, we hypothesized that pre-administration of Z-100 would more strongly suppress tumors.

Here, we tested our theory that pre-administration of Z-100 would strongly suppress tumors. Once confirmed, we examined the effect of Z-100 on the tumor microenvironment under these conditions. Using this model, we further examined the mechanism of the antitumor effects of Z-100 using an anti-CD8 antibody and IL-12p40 KO mice.

## Materials and methods

### Cell line and animals

The mouse oral squamous cell carcinoma cell line Sq-1979 was obtained from RIKEN BRC (Ibaraki, Japan) and cultured in Minimum Essential Medium Eagle (Sigma-Aldrich, MO, USA) supplemented with 10% fetal bovine serum (Hyclone Laboratories, UT, U.S.A.) and Penicillin–Streptomycin Solution (FUJIFILM Wako Pure Chemical, Osaka, Japan), and kept at 37 °C in 5% CO_2_/95% air. Female C3H/HeN mice purchased from Jackson Laboratory Japan (Yokohama, Japan) were housed in pathogen-free conditions. IL-12p40 knockout (KO) mice were generated and provided by Jackson Laboratory Japan on a C3H/HeN background. IL-12p40 gene knockout was confirmed by polymerase chain reaction (PCR)-based genotyping. All mouse experimental protocols were approved by the Animal Ethics Committee of Zeria Pharmaceutical Co., Ltd.

### Z-100

Z-100 was produced by Zeria Pharmaceutical Co., Ltd. (Tokyo, Japan), and diluted in physiological saline for in vivo administration or use in vitro. Z-100 and physiological saline were administered at 5 mL/kg.

### Tumor model

Saline or Z-100 was administered subcutaneously into the right inguinal region of mice once daily for 49 days. On the 29th day of administration of saline or Z-100, Sq-1979 cells (1 × 10^6^ cells/mouse) were injected into the right flank. Tumor growth was monitored by measuring the tumor size (mm^2^) using a caliper once a week. Tumor size was calculated using the formula: long diameter (mm) × short diameter (mm) of tumors. Survival time of mice was recorded up to 200 days after tumor injection.

In the anti-CD8 antibody experiment, 1 mg/mL control IgG (Bio X Cell, NH, USA) or 1 mg/mL anti-CD8α antibody (Bio X Cell) dosing solution was administered intraperitoneally (0.1 mL/body) every 7 days from 3 days before cell injection until 46 days after cell injection. On day 49, mice were euthanized and left inguinal lymph nodes were collected and homogenized to prepare single cell solutions. The dose and dosing interval of the test antibody were selected based on the regimen that showed sufficient CD8^+^ T cell loss in a preliminary study (Additional file 1: Figure S1).

In the tumor-infiltrating cells experiment, tumors and right inguinal lymph nodes were collected 7 days after injection of Sq-1979 cells. Five tumors or lymph nodes from different animals were pooled into a single tube to make one sample. Tumor infiltrating cells were isolated from tumors using a Tumor Dissociation kit (Miltenyi Biotec, Cologne, Germany) according to the manufacturer’s instructions.

### Flow cytometric analysis

Before immunofluorescence staining of cells, Fc receptors were blocked using FcR Blocking Reagent (Miltenyi Biotec). Subsequently, the cells were stained with fluorochrome-labeled mouse antibodies and analyzed using a MACSQuant^®^ Analyzer (Miltenyi Biotec). Dead cells identified by propidium iodide (Miltenyi Biotec) staining were excluded from the analysis. All antibodies were purchased from Miltenyi Biotec except where noted. The following antibodies were used to stain tumor infiltrating CD8^+^ T cells: CD45-VioBlue, CD4-FITC, CD8a PE-Cy7 (Thermo Fisher Scientific, MA, U.S.A.), MHC class II-APC and APC/Cy7-TCRβ (BioLegend, CA, U.S.A.). The following antibodies were used for detailed analysis of CD8^+^ T cells: CD45-VioBlue, CD4-VioGreen, CD44-FITC, CD8a PE-Cy7 (Thermo Fisher Scientific), CD25-APC, CD69-APC-Vio770 and CD62L-PE. The following antibodies were used in the anti-CD8 antibody experiment: CD45-VioBlue, CD8a-PE-Vio770 and APC/Cy7-TCRβ (BioLegend).

### Measurement of ATP levels in Sq-1979 cells

Sq-1979 cells (2000 cells/well) were cultured for 24 h in a 96-well cell culture plate. Thereafter, saline (control), Z-100 (0.1, 1, 10 or 100 μg/mL) or cisplatin (30 μmol/L, FUJIFILM Wako Pure Chemical) was added to the wells in triplicate. Twenty-four hours after addition of the test compounds, cell viability in each well was assayed by measuring the luminescence in relative light units (RLU) using CellTiter-Glo^®^ Luminescent (Promega, WI, USA) according to the manufacturer’s instructions. This experiment was repeated 6 times.

### Measurement of IL-12p40 in bone marrow-derived macrophages (BMDM)

Female C3H/HeN mice were sacrificed by cervical dislocation and bone marrow cells were collected. The bone marrow cells were cultured in RPMI1640 medium (Thermo Fisher Scientific) containing 10% fetal bovine serum (Hyclone Laboratories), 200 U/mL penicillin (Meiji Seika Pharma, Tokyo, Japan), 200 µg/mL streptomycin (Meiji Seika Pharma) and 20 ng/mL murine GM-CSF (PeproTech, NJ, U.S.A.) at 37 °C in 5% CO_2_/95% air. On the second day of culture, 75% of the culture medium was replaced, and on the fourth day, all of the medium was replaced. On the seventh day, cells adhering to the dish were collected with a scraper. The cell suspension (2 × 10^5^/100 µL/well) was added to 96-well plates and incubated with 20 ng/mL murine GM-CSF for 30 min. Thereafter, the culture supernatant was removed and each stimulant was added to the above medium containing 0.3 ng/mL LPS without GM-CSF. After 24 h, the culture supernatant was collected and IL-12p40 production was measured using an ELISA kit (R&D Systems, MN, U.S.A.) according to the manufacturer’s instructions.

### Statistical analysis

*P*-values less than 0.05 were considered statistically significant. To assess differences between tumor growth curves among the four groups, *P*-values were calculated by repeated measures ANOVA with Dunnett’s multiple comparisons test; to assess differences between two groups, repeated measures ANOVA was used. To assess differences in survival curves between saline- and Z-100-treated groups, *P*-values were calculated using the log-rank test. For ATP levels, the homoscedasticity of the 6 groups was first evaluated using Bartlett’s test. As the 6 groups were determined to be homoscedastic, the *P*-value was calculated using parametric Dunnett’s multiple comparisons test. In all other cases, the homoscedasticity of the groups was first evaluated using an F-test. When the groups were homoscedastic, the *P*-value was calculated using Student’s t-test. When the groups were not homoscedastic, the *P*-value was calculated using Aspin-Welch’s t-test.

## Results

### Antitumor effect of Z-100 on Sq-1979 tumor model

**Fig. 1:**
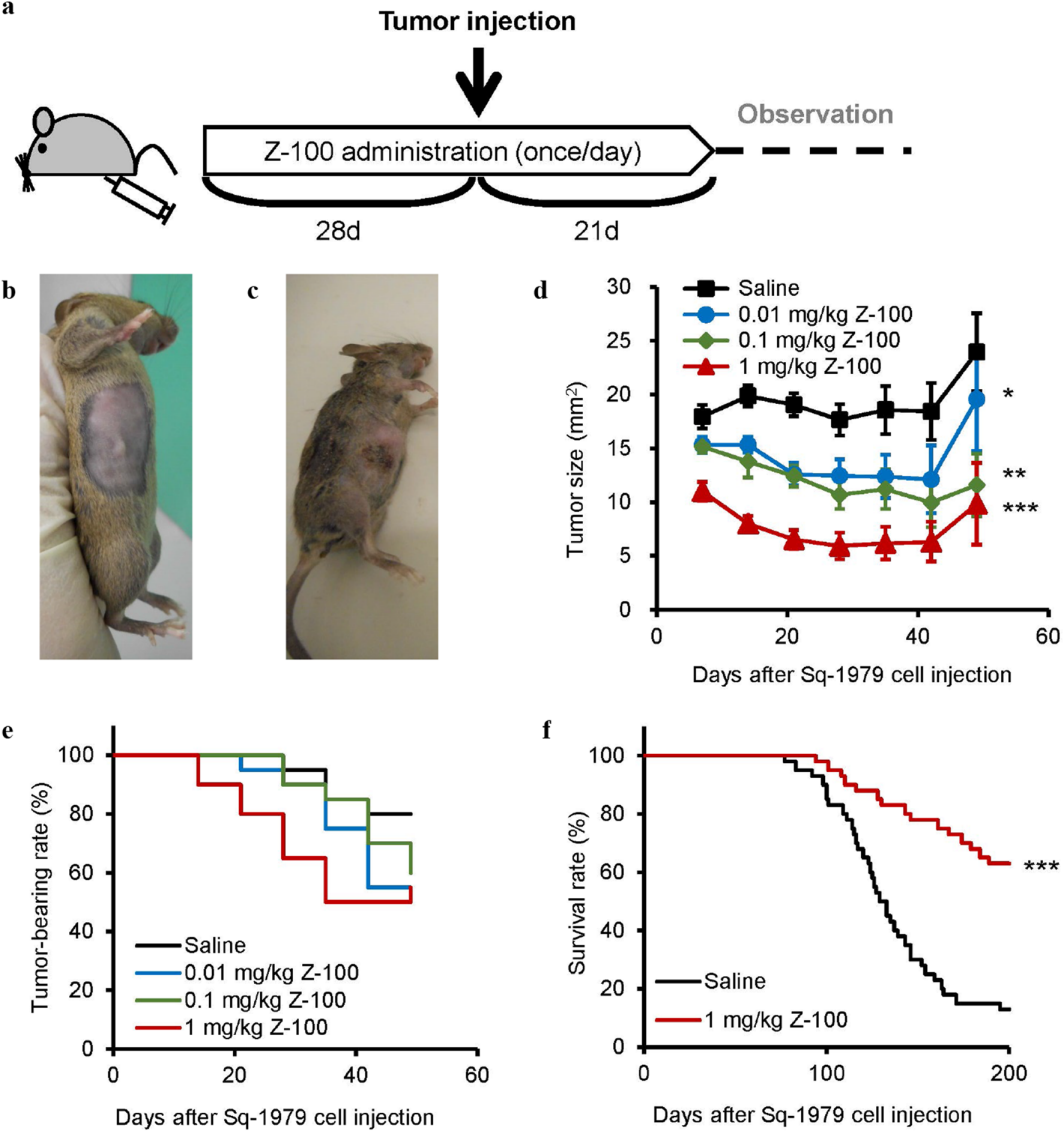
Antitumor effect of Z-100 in Sq-1979 model. **a** Schematic of the experimental design. Saline or Z-100 was administered subcutaneously into the right inguinal region of female C3H/HeN mice once daily for 49 days. On the 29th day of administration of saline or Z-100, Sq-1979 cells (1 × 10^6^ cells) were injected subcutaneously into the right flank of the mice. Photographs of tumor model mice at 1 week after Sq-1979 cell injection (**b**) and at death (**c**). **d** Tumor growth in the Saline or Z-100 (0.01, 0.1, or 1 mg/kg)-administered groups (n = 20). Tumor growth was monitored by measuring tumor size (mm^2^) using a caliper. Data show mean ± SE; n = 20 per group. Asterisks indicate a significant difference compared with the Saline group; * P < 0.05, ** P < 0.01 and *** P < 0.001 (repeated measures ANOVA with Dunnett’s multiple comparisons test). **e** Tumor-bearing rate. **f** Survival rate in the Saline or 1 mg/kg Z-100-administered group (n = 40). Survival time of mice was recorded for up to 200 days after tumor injection and used to generate a survival curve. Asterisks indicate a significant difference compared with the Saline group; *** P < 0.001 (log-rank test)

Although Z-100 has been examined in clinical trials for squamous cell carcinoma of the uterine cervix [17, 18], there is currently no widely used mouse cervical cancer cell line. Therefore, we used Sq-1979, a mouse oral squamous cell carcinoma cell line. Z-100 was subcutaneously administered at doses of 0.01, 0.1 or 1 mg/kg to a tumor model established by subcutaneous injection of Sq-1979 as shown in Fig. 1a. Figure 1b, c show photographs of Sq-1979 tumor model mice at 1 week after Sq-1979 cell injection and at death. Z-100 suppressed tumor growth in a dose-dependent manner and reduced the tumor-bearing rate (Fig. 1d, e). Furthermore, we found that 1 mg/kg Z-100 increased survival (Fig. 1f) while significantly reducing tumor size in this model (Additional file 2: Figure S2). It should be noted that we compared survival between only the Saline group and the 1 mg/kg Z-100 group because our preliminary studies indicated that 40 animals per group were required to confirm the drug’s effect on lifespan (data not shown), and our limited sample size did not allow for further comparisons.

**Fig. 2:**
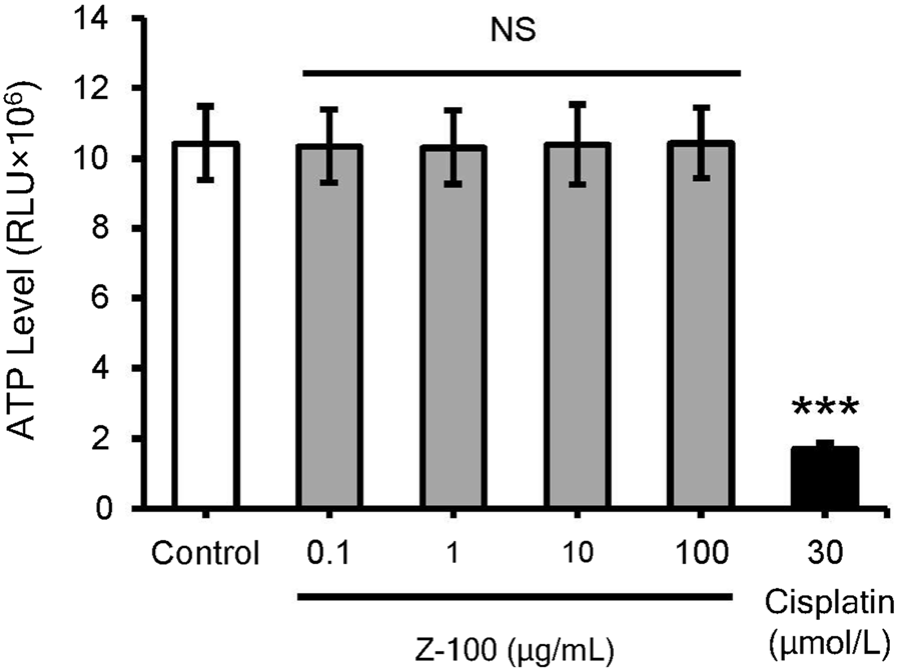
Direct effect of Z-100 on Sq-1979 cell line. Cell viability of Sq-1979 stimulated with saline (control), Z-100 (0.1, 1, 10 and 100 μg/mL) or cisplatin (30 μmol/L). After culturing Sq-1979 cells (2000 cells/well) for 24 h in a 96-well plate, test compounds were added to the wells. Twenty-four hours after addition of the test compounds, ATP levels in each well were assayed by measuring the luminescence in relative light units (RLU). Data show mean ± SD; n = 6 (triplicate experiment repeated 6 times). Asterisks indicate a significant difference compared with the Control group; *** P < 0.001, NS, not significant (parametric Dunnett’s multiple comparisons test)

Next, to examine whether Z-100 induces direct killing of Sq-1979 cells, we checked the ATP levels of Sq-1979 cells after addition of Z-100 in vitro (Fig. 2). Cisplatin, which was used as a positive control, decreased ATP levels in Sq-1979 cells, but Z-100 did not.

### *Z-100-mediated tumor infiltration of CD8*^+^*T cells*

**Fig. 3:**
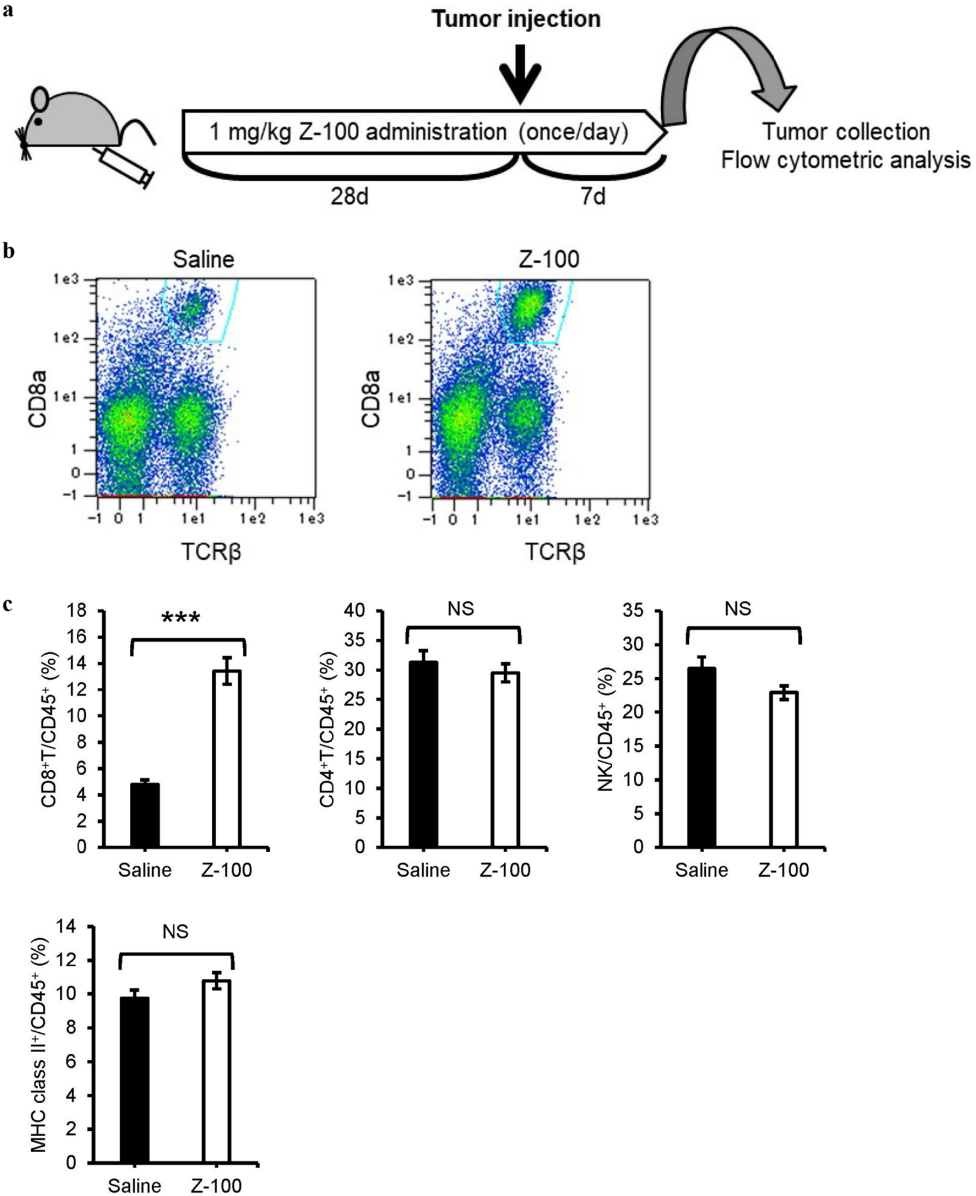
Effect of Z-100 on tumor infiltrating cells. **a** Schematic of the experimental design. Saline or Z-100 (1 mg/kg) was administered subcutaneously into the right inguinal region of female C3H/HeN mice once daily for 49 days. On the 29th day of administration of saline or Z-100, Sq-1979 cells (1 × 10^6^ cells) were injected subcutaneously into the right flank of the mice. Seven days after injection of Sq-1979, tumors were collected and analyzed by flow cytometry. **b** Typical dot plots of CD8^+^ T cells among CD45^+^ cells. **c** Ratio of tumor infiltrating cells. Data show mean ± SE; n = 8 (pool of 5 animals per sample). Asterisks indicate a significant difference compared with the Saline group; *** P < 0.001 (Aspin-Welch’s t-test), NS, not significant (Student’s t-test)

To confirm the effects of Z-100 on immune cells, we used flow cytometry to identify the tumor infiltrating cells in our model. As shown in Fig. 1e, the tumors of some mice in the 1 mg/kg Z-100 group had disappeared by 14 days after tumor injection. Therefore, tumors were collected 7 days after tumor injection, and tumor infiltrating cells were isolated and analyzed (Fig. 3a). Administration of 1 mg/kg Z-100 increased the ratio of CD8^+^ T/CD45^+^ tumor infiltrating cells (Fig. 3b, c). In contrast, Z-100 did not significantly change the ratio of CD4^+^ T cells, NK cells and MHC class II^+^ to CD45^+^ cells. These results suggest that Z-100 induces the infiltration of CD8^+^ T cells into tumors.

### *Effector memory cells in tumor-infiltrating CD8*^+^*T cells*

**Fig. 4:**
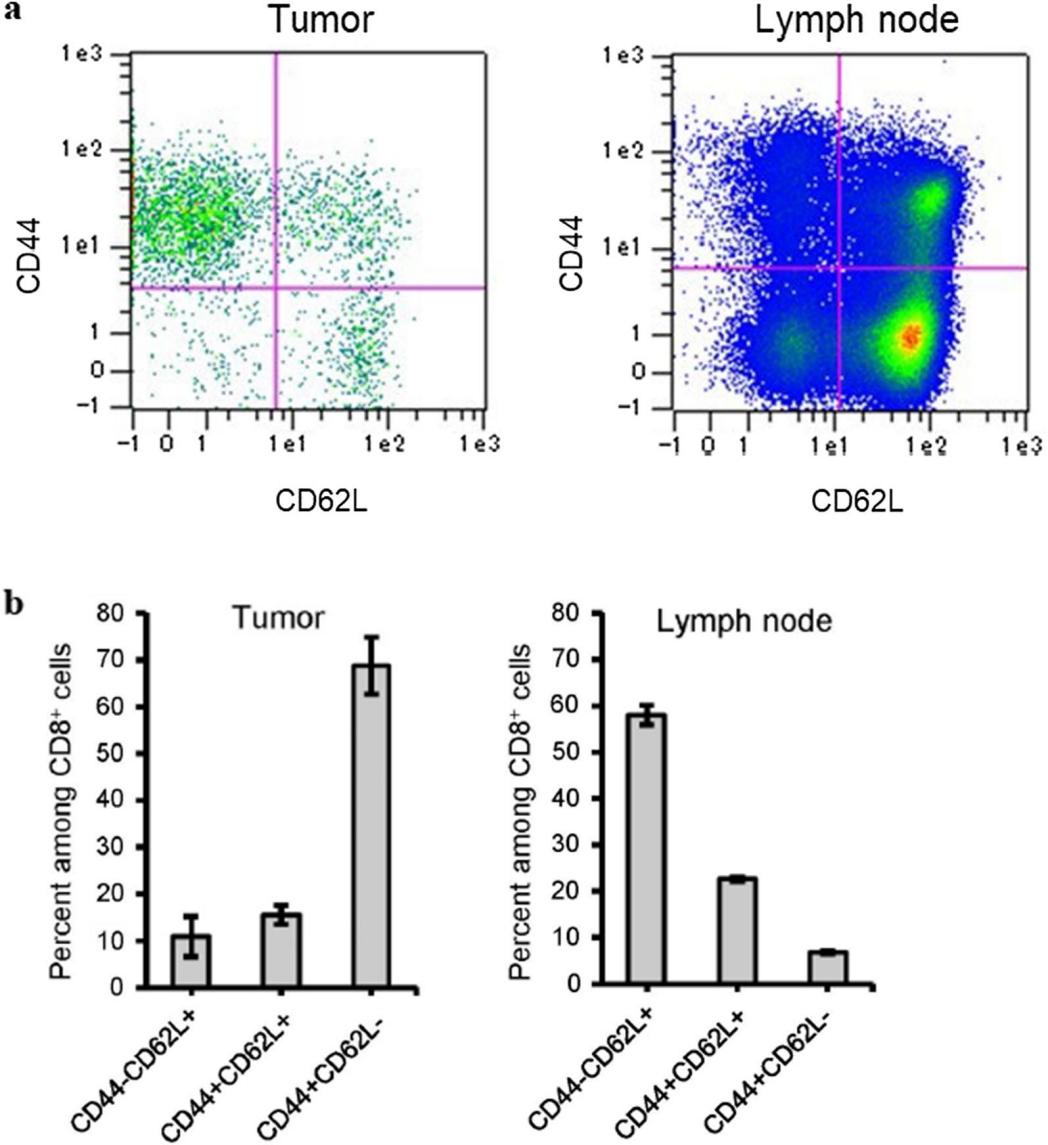
Detailed analysis of tumor infiltrating CD8^+^ cells. Z-100 (1 mg/kg) was administered subcutaneously into the right inguinal region of female C3H/HeN mice once daily for 49 days. On the 29th day of administration of Z-100, Sq-1979 cells (1 × 10^6^ cells) were injected subcutaneously into the right flank of the mice. Seven days after injection of Sq-1979, tumors and right inguinal lymph nodes were collected and analyzed by flow cytometry. **a** Typical dot plots of cells expressing CD44 and CD62L among CD8^+^ cells. **b** Ratio of each type of cell among CD8^+^ cells. Data show mean ± SE; n = 8 (pool of 5 animals per sample)

Next, we conducted a more detailed analysis of the CD8^+^ T cells identified in tumors following Z-100 administration. Similar to the experiment presented in Fig. 3a, tumors and inguinal lymph nodes of mice administered 1 mg/kg Z-100 were collected and CD8^+^ cells within these tissues were checked for CD44 and CD62L expression. We found that most of the CD8^+^ cells in tumors were CD44^+^CD62L^−^ cells (Fig. 4), which are known to be effector memory cells [19, 20]. In contrast, many CD44^−^CD62L^+^ cells were found in the lymph nodes (Fig. 4), which are known to be naive cells [19, 20]. Although we also attempted to conduct detailed analysis of the tumor-infiltrating cells in the Saline group, the number of cells obtained was insufficient to provide reliable data.

### Abolishment of the antitumor effects of Z-100 by anti-CD8 antibody

**Fig. 5:**
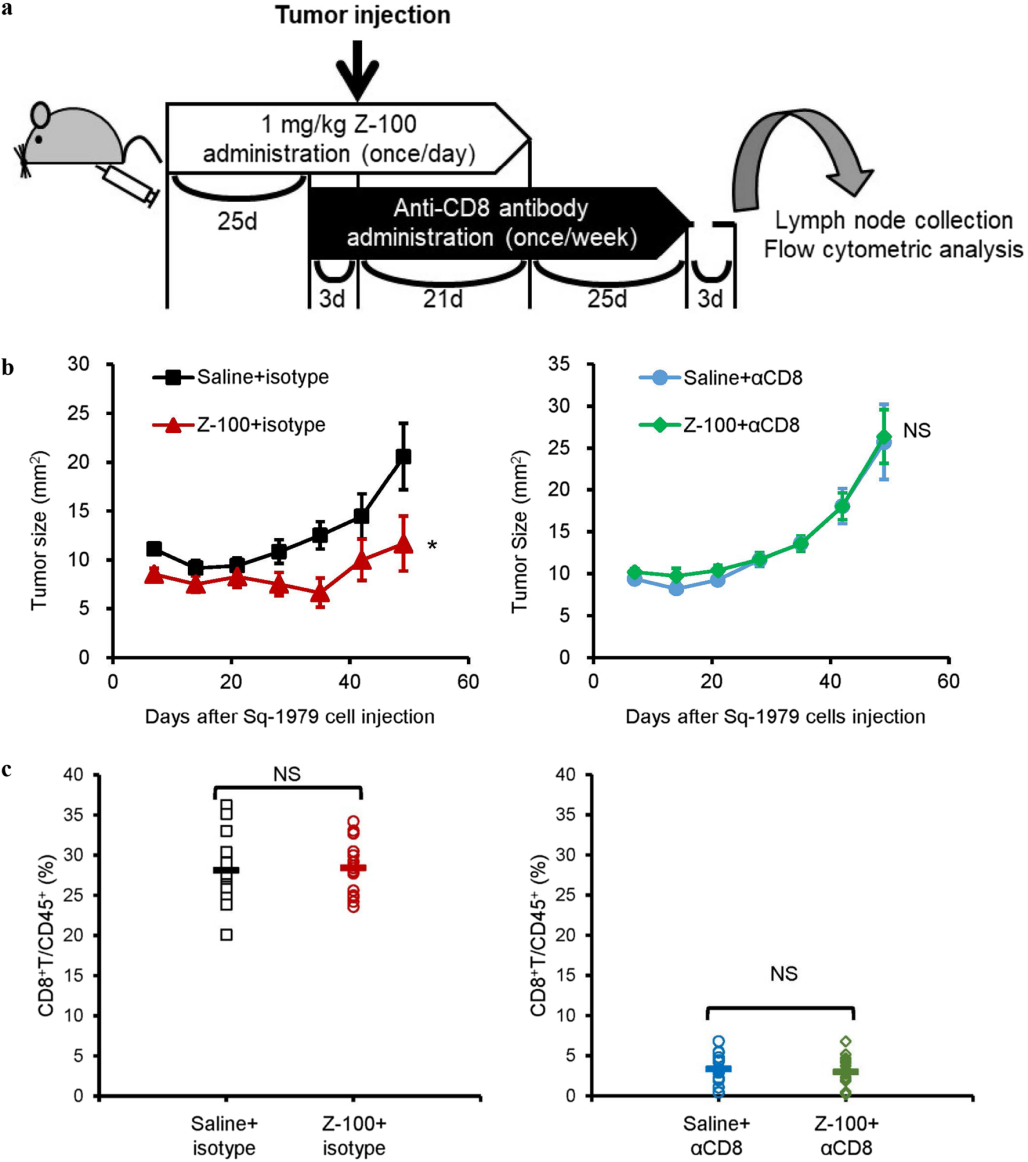
Abolishment of the antitumor effects of Z-100 by anti-CD8 antibody. **a** Schematic of the experimental design. Saline or Z-100 (1 mg/kg) was administered subcutaneously into the right inguinal region of C3H/HeN female mice once daily for 49 days (n = 18–20). On the 29th day of saline or Z-100 administration, Sq-1979 cells (1 × 10^6^ cells) were injected into the right flank. Moreover, control IgG (0.1 mg/body) or anti-CD8 antibody (0.1 mg/body) was administered intraperitoneally every 7 days from 3 days before cell injection until 46 days after cell injection. **b** Tumor growth in the Saline- or Z-100-administered group with antibody. Tumor growth was monitored by measuring tumor size (mm^2^) using a caliper. Data show mean ± SE. Asterisks indicate a significant difference compared with the Saline group; * P < 0.05 (repeated measures ANOVA). **c** Ratio of CD8^+^T/CD45^+^ in lymph nodes. Symbols indicate individual ratios and horizontal bars indicate mean ratios. NS, not significant (Student’s t-test)

Given that Z-100 administration increased CD8^+^ T cells in tumors, we subsequently examined whether removing CD8^+^ T cells could abolish the antitumor effects of Z-100. As shown in Fig. 5a, intraperitoneal administration of an anti-CD8 antibody was started 3 days prior to injection of Sq-1979 cells to remove CD8^+^ T cells. We found that administration of the anti-CD8 antibody abolished the antitumor effects of Z-100 (Fig. 5b). This result suggests that CD8^+^ T cells contribute to the antitumor effects of Z-100. We confirmed that CD8^+^ T cells were indeed removed at the end of the experiment by collecting left inguinal lymph nodes and measuring the CD8^+^ T cell ratio using a flow cytometer (Fig. 5c).

### Abolishment of the antitumor effects of Z-100 in IL-12p40 KO mice

**Fig. 6:**
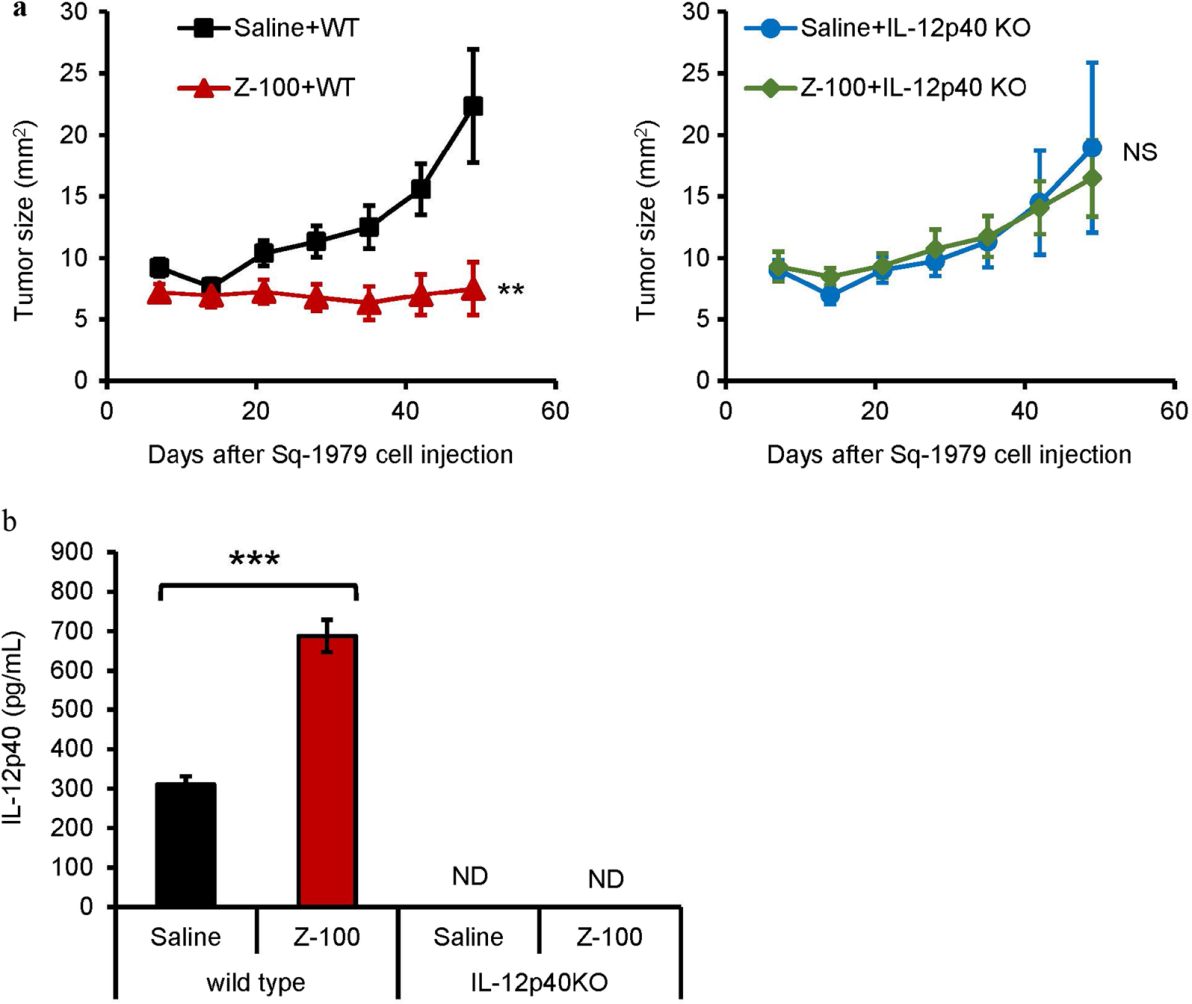
Abolishment of the antitumor effect of Z-100 in IL-12p40 KO mice. **a** Tumor growth in C3H/HeN or IL-12p40 KO mice administered saline or Z-100 (1 mg/kg) (n = 15–20). Saline or Z-100 was administered subcutaneously into the right inguinal region of female C3H/HeN mice or IL-12p40 KO mice once daily for 49 days. On the 29th day of administration of saline or Z-100, Sq-1979 cells (1 × 10^6^ cells) were injected subcutaneously into the right flank of the mice. Tumor growth was monitored by measuring tumor size (mm^2^) using a caliper. Data show mean ± SE. Asterisks indicate a significant difference compared with each Saline group; ** P < 0.01, NS, not significant (repeated measures ANOVA). **b** IL-12p40 production from BMDM derived from C3H/HeN or IL-12p40 KO mice stimulated with saline or Z-100 (100 µg/mL). Bone marrow cells were cultured in the presence of GM-CSF for 7 days to enable them to differentiate into BMDMs, then seeded onto plates and pre-incubated for 30 min. Stimulation was started after pre-incubation. After 24 h, the supernatant was collected and the IL-12p40 concentration in the supernatant was determined by ELISA. Data show mean ± SE. Asterisks indicate a significant difference compared with each Saline group; *** P < 0.001 (Student’s t-test), ND, not detected

We previously reported that Z-100 increases IL-12p40 production [13]. Therefore, we examined whether the antitumor effects of Z-100 were abolished in IL-12p40 KO mice and found that, indeed, the antitumor effects of Z-100 were absent in these animals, as shown in Fig. 6a. We further confirmed that IL-12p40 production was absent in IL-12p40 KO mice (Fig. 6b) by measuring IL-12p40 production from BMDM differentiated from GM-CSF-stimulated bone marrow-derived cells. These findings suggest that Z-100 inhibits tumor growth by increasing IL-12p40 production.

## Discussion

The antitumor effects of Z-100 have been reported in several papers [7–10]. In this study, the antitumor effect of Z-100 was dose-dependent and, although 1 mg/kg was considered the optimal dose, Z-100 significantly inhibited tumor growth even at 0.01 mg/kg. With the exception of one non-clinical study, there have been no previous reports on the antitumor effects of Z-100 at doses as low as 0.01 mg/kg. The lone study that used such low doses of Z-100 examined its antitumor effects on an inguinal lymph node metastasis model [11]. This prior study and the present study are similar in that both used “pre-administration” of Z-100 at low doses. In the study on inguinal lymph node metastasis, Z-100 administration was started at the same time as tumor injection into the plantar region; however, because the tumor did not metastasize into inguinal lymph nodes until 2–3 weeks later, the strategy can be considered “pre-administration” of Z-100 for treatment of cancer metastasis in the inguinal lymph nodes. Therefore, administering Z-100 prior to cancer exacerbation may be key to maximizing its effects. While it is not possible to prophylactically administer medicines against cancer development in clinical practice, it is possible to “pre-administer” them to prevent metastasis, recurrence or relapse. Thus, Z-100 may be clinically effective for inhibiting malignant transformation of cancer.

Our in vivo experimental system, in which Z-100 showed high efficacy, made it possible to investigate tumor infiltrating cells, leading to the identification of infiltrating CD8^+^ T cells. Further analysis showed that these infiltrating CD8^+^ T cells were effector memory cells, and that an anti-CD8 antibody abolished the antitumor effects of Z-100. Thus, there is no doubt that the antitumor effects of Z-100 are mediated by CD8^+^ T cell infiltration. That administration of Z-100 increased the proportion of infiltrating CD8^+^ T cells suggests that the tumors had been converted to hot tumors, and that Z-100 could be a hot tumor inducer. Immunotherapies that include an anti-PD-1 antibody have been reported to be highly effective against hot tumors [1, 4, 21], which may explain why the combination of Z-100 and anti-PD-1 antibody was effective in the report by Kobayashi et al. [14]. Further, we previously reported that Z-100 increases IL-12p40 production [13]. IL-12p40 is known bind to IL-12p35 to form the heterodimer IL-12, which is involved in antitumor immunity [22]. Some studies have suggested that inducing IL-12 leads to the formation of hot tumors [23, 24], while another report indicated that suppressing IL-12p40 leads to cold tumors [25]. Therefore, it is possible that the inability of Z-100 to increase IL-12p40 production in IL-12p40 KO mice may have prevented tumors from becoming hot tumors. However, it is questionable whether administration of IL-12 would have the same effect as administration of Z-100. Although administration of IL-12 has been reported to have anti-tumor effects, toxicity has also been observed [26–30]. Given that Z-100 has very mild toxicity in clinical practice [17], its effects may differ from those of IL-12 administration. Z-100 stimulation has been reported to increase TNF-α, IL-1β, IFN-γ, and IL-2 from immune cells [9, 10, 13, 31], the production of which may underlie the different activities of Z-100 and IL-12. We also previously reported that Z-100 increases IL-12p40 production from M1-like macrophages differentiated from mouse bone marrow cells and human CD14^+^ cells in the presence of GM-CSF [13]. In that report, peptidoglycans were present in the Z-100, and commercially available peptidoglycans have been shown to increase IL-12p40 production from these cells. Further, knockdown of nucleotide-binding oligomerization domain 2 (NOD2) suppressed this Z-100-mediated increase in IL-12p40 production. Therefore, we concluded that the peptidoglycans in Z-100 led to production of IL-12p40 via NOD2. Thus, peptidoglycans from *Mycobacterium tuberculosis* and NOD2 agonists could also be hot tumor inducers. Further studies are needed to confirm this theory.

In this study, we used the oral squamous cell carcinoma cell line Sq-1979. In general, squamous cell carcinoma is known as a hot tumor-like cancer, while melanoma is known as a cold tumor-like cancer [32]. In a previous study, Z-100 suppressed tumor growth and increased survival in a subcutaneous injection model of B16 melanoma [10]. Z-100 has also shown an inhibitory effect on lung metastasis models of B16 melanoma [9]. In addition, Z-100 suppressed metastasis in a lymphatic metastatic model of B16 melanoma and increased the number of immune cells in lymph nodes [11]. These reports suggest that Z-100 is also effective against cold tumors.

In summary, this study found that "pre-administration" of Z-100 induced potent antitumor effects, and that Z-100 acts by inducing the infiltration of CD8^+^ T cells into tumors. This suggests that Z-100 may convert tumors into hot tumors for more favorable response to immunotherapy. Thus, Z-100 is expected to have antitumor effects in clinical practice, including in combination with various immunotherapies.

## Supplementary Information

**Additional file 1: Figure S1.** Examination of antibody dose and time to disappearance of CD8^+^ T cells.

**Additional file 2: Figure S2.** Tumor size and tumor-bearing rate.

## Data Availability

The data that support the findings of this study are available from the corresponding author upon reasonable request.
