# Supplementary material for: Potential of Z-100, extracted from Mycobacterium tuberculosis strain Aoyama B, as a hot tumor inducer
Source: Cancer Cell Int. 2022 Dec 9;22:392. doi: 10.1186/s12935-022-02821-6 (PMC9733245; doi:10.1186/s12935-022-02821-6)
Supplement: Supplementary file 1 — Additional file 1: Figure S1. Examination of antibody dose and time to disappearance of CD8+ T cells. [file 12935_2022_2821_MOESM1_ESM.docx]

**a
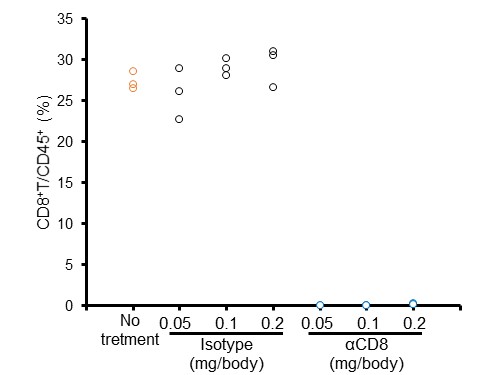
**

**b
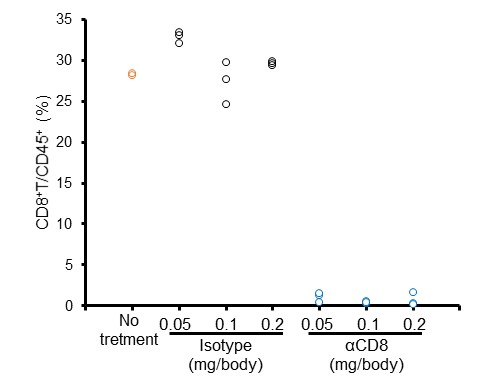
**

**Figure S1: Examination of antibody dose and time to disappearance of CD8^+^ T cells**

C3H/HeN mice were administered a single intraperitoneal dose of control IgG (0.05, 0.1 or 0.2 mg/body) or anti-CD8 antibody (0.05, 0.1 or 0.2 mg/body). Left inguinal lymph nodes were collected from mice 4 or 7 days after antibody administration and analyzed by flow cytometry. Symbols indicate individual ratios. (a) CD8^+^T/CD45^+^ cell ratio in lymph nodes 4 days after antibody administration. (b) CD8^+^T/CD45^+^ cell ratio in lymph nodes 7 days after antibody administration.
