# Supplementary material for: Potential of Z-100, extracted from Mycobacterium tuberculosis strain Aoyama B, as a hot tumor inducer
Source: Cancer Cell Int. 2022 Dec 9;22:392. doi: 10.1186/s12935-022-02821-6 (PMC9733245; doi:10.1186/s12935-022-02821-6)
Supplement: Supplementary file 2 — Additional file 2: Figure S2. Tumor size and tumor-bearing rate. [file 12935_2022_2821_MOESM2_ESM.docx]

**a b**


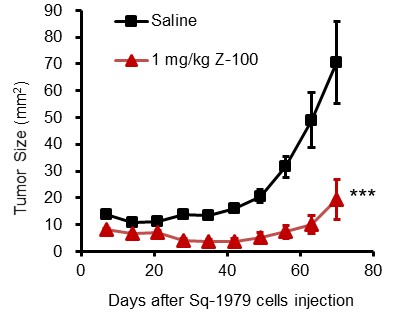

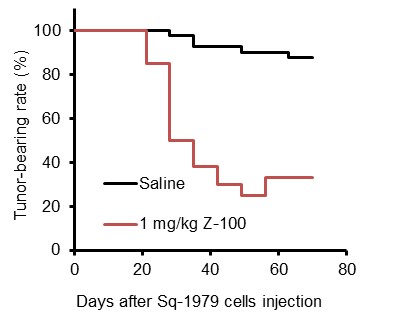


**Figure S2: Tumor size and tumor-bearing rate**

Tumor size and tumor-bearing rate in the same animals used to determine survival rate in Figure 1d (n = 40). Data show mean ± S.E. Asterisk indicates a significant difference compared with the Saline group; *** P<0.001 (repeated measures ANOVA).
